# Six-Year Incidence and Progression of Age-Related Macular Degeneration in Kenya: Nakuru Eye Disease Cohort Study

**DOI:** 10.1001/jamaophthalmol.2017.1109

**Published:** 2017-06-08

**Authors:** Andrew Bastawrous, Wanjiku Mathenge, Tunde Peto, Nisha Shah, Kevin Wing, Hillary Rono, Helen A. Weiss, David Macleod, Allen Foster, Matthew Burton, Hannah Kuper

**Affiliations:** 1 International Centre for Eye Health, Clinical Research Department, London School of Hygiene & Tropical Medicine, London, England; 2 Rwanda International Institute of Ophthalmology and Dr Agarwal’s Eye Hospital, Kigali, Rwanda; 3 Department of Non-communicable Disease Epidemiology, London School of Hygiene & Tropical Medicine, London, England; 4 Centre for Public Health, Queen’s University Belfast, United Kingdom; 5 National Institute for Health Research Biomedical Research Centre at Moorfields Eye Hospital National Health Service Foundation Trust, London, England; 6 University College London Institute of Ophthalmology, London, England; 7 Kitale Eye Unit, Kitale, Kenya; 8 Medical Research Council Tropical Epidemiology Group, Department of Infectious Disease Epidemiology, London School of Hygiene & Tropical Medicine, London, England; 9 Moorfields Eye Hospital National Health Service Foundation Trust, London, England

## Abstract

**Importance:**

The incidence of age-related macular degeneration (AMD) is unknown in Africa.

**Objective:**

To estimate the 6-year cumulative incidence and progression of AMD in older adults (≥50 years old) in Nakuru, Kenya.

**Design, Setting, and Participants:**

This study assessed a population-based cohort with 6-year follow-up of 4414 participants who had a complete assessment. Random cluster sampling with probability proportionate to size procedures was used to select a representative, cross-sectional sample of adults 50 years and older from January 26, 2007, through November 11, 2008. A 6-year follow-up was undertaken from January 7, 2013, through March 12, 2014. On both occasions, a comprehensive ophthalmic examination was performed that included logMAR visual acuity, digital retinal photography, and grading of images at Moorfields Eye Hospital Reading Centre. Data were collected on general health and risk factors.

**Main Outcomes and Measures:**

Incident AMD in participants with no AMD at baseline and progression from early to late AMD.

**Results:**

A total of 1453 of the 2900 individuals (50.1%) at risk for AMD were followed up after 6 years (mean [SD] age, 60.7 [8.2] years; 635 female [49.5%]; 799 Kikuyu [62.3%], 324 Kalenjin [25.3%], and 159 other [12.4%]); 1282 had data on AMD status at follow-up. Of these, 202 developed early AMD, and no participants developed late AMD. The 6-year weighted (for loss to follow-up) cumulative incidence of early AMD was 164.2 per 1000 persons (95% CI, 136.7-195.9 per 1000 persons). Two individuals with baseline early AMD from the 142 at risk had developed late AMD at follow-up, with a 6-year cumulative incidence of progression from early to late AMD of 24.5 per 1000 persons (95% CI, 5.0-111.7 per 1000 persons). Cumulative incidence of AMD increased with age (≥80 years old vs 50-59 years old: 1.8; 95% CI, 0.9-3.5) and was higher in women (female vs male: 1.6; 95% CI, 1.2-2.1) and persons with diabetes (diabetes vs no diabetes: 1.7; 95% CI, 1.0-2.8).

**Conclusions and Relevance:**

In Kenya, more than 100 000 estimated new cases of AMD, mainly early AMD, will develop every year in individuals 50 years or older, although a 50% loss to follow-up and wide CIs for progression to late AMD limit definitive conclusions from these findings.

## Key Points

**Question:**

What is the incidence of age-related macular degeneration in Kenya?

**Findings:**

A 6-year, population-based cohort study of 4414 adult Kenyans (≥50 years of age) was conducted, and the 6-year weighted cumulative incidence of early age-related macular degeneration was 164.2 per 1000 persons.

**Meaning:**

These results suggest that age-related macular degeneration may become a greater public health concern in Kenya and similar countries in the future with population aging in these regions.

## Teaser

This population-based cohort study estimates the 6-year cumulative incidence and progression of age-related macular degeneration in older adults (≥50 years of age) in Nakuru, Kenya.

## Introduction

Age-related macular degeneration (AMD) is a progressive degenerative disease that affects the central retina and is highly associated with age.^1^ Advanced AMD, including geographic atrophy (late dry) and neovascular AMD (wet), leads to central vision loss. In the early dry form of the disease, deposits known as drusen are layered between the retina and choroid, and subtypes of drusen (based on size and morphologic features) form part of the more detailed classifications. Although AMD is a leading cause of visual impairment and blindness in populations living in high-income countries,^2^ there is a paucity of available data from low- and middle-income countries,^3^ including sub-Saharan Africa. However, a systematic review^4^ found that overall posterior-segment disease is a common cause of visual impairment in sub-Saharan Africa, and a survey^5^ in Kenya found that 1 in 10 persons 50 years and older had signs of AMD.

Estimation of the incidence and progression of AMD and associated sight loss is important for planning of services. Treatment of neovascular AMD is currently possible in well-established health care systems but infrequently available in low- and middle-income countries. It is therefore important to be able to identify individuals at high risk for AMD to consider targeted approaches for prevention and/or treatment. Furthermore, rehabilitation services need to be planned for individuals developing visual loss as a result of AMD. Unfortunately, data to plan these services are currently lacking. The incidence of AMD has been investigated in 7 cohort studies of eye disease worldwide,^6^ with no data from the African continent. There are large variations in the prevalence, phenotypes, and incidence of AMD in different populations,^6^ making extrapolation of findings from studies in other regions of the world to an African setting difficult. The aims of the current study were to estimate the 6-year cumulative incidence of AMD in Nakuru, Kenya, and to identify risk factors for incident disease.

## Methods

We studied a population-based cohort with 6-year follow-up of 4414 participants who had a complete assessment. Random cluster sampling with probability proportionate to size procedures was used to select a representative, cross-sectional sample of adults 50 years and older from January 26, 2007, through November 11, 2008. A 6-year follow-up was undertaken from January 7, 2013, through March 12, 2014. The following examination protocols were implemented at baseline and follow-up, with detailed methods available elsewhere^17^ and in the eMethods in the Supplement. The London School of Hygiene & Tropical Medicine Ethics Committee and the African Medical Research Foundation granted ethical approval for the study, which was also approved by the provincial medical officer for Nakuru County. Written approval was sought from the administrative heads in each cluster, usually the village chief. All participants gave written or thumbprint consent to participate. People requiring medical treatments were referred to the appropriate center. All data were deidentified.

### Ophthalmic and General Examination

All participants underwent logMAR visual acuity testing on each eye separately and corrected visual acuity when less than 20/40 Snellen equivalent. Detailed interviews were undertaken in the local language on demographic details, information on risk factors, socioeconomic status, and full medical history. A nurse recorded the blood pressure, weight, height, and waist and hip circumferences. Participants had 2 nonstereoscopic, digital, 45° fundus photographs (1 disc and 1 macula centered) taken per eye by an ophthalmic clinical officer. Digital images were graded at an approved grading center. The senior grader (N.S.) graded all images for the presence of AMD. All eyes classified as having late-stage AMD were adjudicated by the Moorfields Eye Hospital Reading Centre clinician (T.P.). The adjudicator (T.P.) also graded 5% of randomly selected images to ensure quality control.

### Definitions of AMD Used

A modified version of the international classification and grading system for age-related maculopathy and AMD was used for image grading at baseline and follow-up.^18^ Drusen were categorized based on size, uniformity of color, and margins. Patients were classified into hard or soft drusen categories: small drusen (<63 μm) were considered to be hard. Large drusen with a uniform density, sharp margins, and a nodular surface texture were placed in the soft distinct category, whereas those without sharp margins were classified as indistinct. When end-stage disease was apparent, patients were classified as having geographic atrophy in the presence of well-demarcated regions with diameters greater than 175 μm, within which large choroidal vessels were clearly visible to the atrophy of the overlying choriocapillaris and retinal pigment epithelium. Neovascular AMD was graded as present when exudative features, such as serous fluid, hemorrhage, lipid exudates, or fibrosis, were seen to be originating primarily from the subretinal, pigment, and epithelial tissue layers.

Case definitions were based on the eye with more severe status if both eyes were gradable and on the gradable eye if only one was gradable. Early AMD was defined as the presence of large, soft drusen and pigmentation greater than 63 μm, and late AMD was defined as the presence of geographic atrophy or neovascular AMD.

Incident AMD was defined on the basis of the absence of AMD features at baseline on retinal images and the subsequent presence of these features at follow-up. Incident late AMD was defined as the combination of no or early AMD at baseline and signs of late AMD at follow-up.

### Dealing With Loss to Follow-up

Logistic regressions corrected for the survey design were used to calculate *P* values to assess differences between participants seen and lost to follow-up and those known to have died. An inverse probability weighting (IPW) model was used to allow estimation of cumulative incidence while accounting for those lost to follow-up. Those who had died between baseline and follow-up were excluded from the analysis. Multivariable logistic regression was used to identify independent baseline covariates associated with loss to follow-up. Covariates for which there was evidence of association with the outcome (*P* < .10) were kept in a multivariable model. Individuals without a complete set of the baseline covariates included in the final multivariable model were excluded from any estimations based on the weighted analysis. From this final model, the probability of being followed up was estimated based on the presence or absence of each of these baseline covariates. The inverse of this probability formed the weighting to be applied to account for those lost to follow-up.

The final step was to exclude those individuals lost to follow-up from the analysis and apply the IPW to account for those lost to follow-up. A sensitivity analysis for this approach involved a complete records analysis (ie, including only individuals who had complete records for outcome and all variables in the analysis).

### Cumulative Incidence Estimation

The 6-year cumulative incidence of AMD was estimated by dividing the total (weighted) number of individuals who were classified as having AMD at follow-up by the (weighted) number of individuals who were AMD free at baseline and examined at follow-up. The 6-year cumulative incidence was then used to estimate the expected number of new AMD cases per year. The size of the at-risk population in Kenya was estimated using the baseline prevalence of AMD from this cohort and the 2015 Kenyan population estimates for those 50 years or older. The 6-year incidence was then multiplied by this at-risk population and divided by 6, with the assumption that cumulative incidence was constant over time. Annual cumulative incidence was also estimated separately for men and women and in 10-year age categories (50-59, 60-69, 70-79, and ≥80 years). The incidence of progression from early to late AMD was calculated by examining participants with early AMD at baseline who were followed up and had a valid AMD status at follow-up.

### Assessing Risk Factors Associated With AMD Incidence

The age-adjusted association between AMD incidence and each covariate was estimated using a Poisson regression model. A multivariable model was created with backward stepwise selection using the likelihood ratio test and a threshold of 2-tailed *P* < .05 for retention of a variable in the model.

## Results

At baseline, 4414 participants had a complete assessment, of whom 3304 (74.9%) had an AMD assessment from retinal imaging (Figure). Of these participants, 404 (12.2%) had AMD at baseline, with 366 (90.6%) having early AMD and 38 (9.4%) having late AMD. An additional 2900 participants did not have AMD at baseline and were therefore at risk for developing AMD at follow-up.^5^

**Figure.**
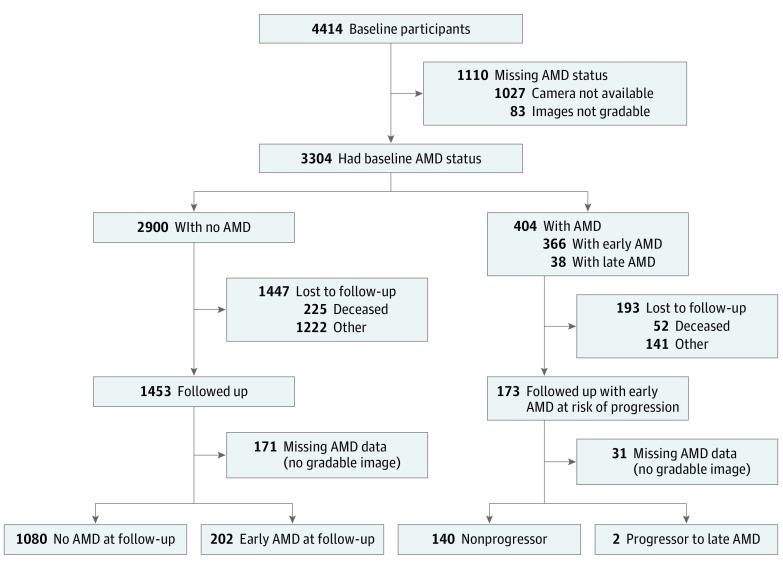
Participant Flowchart AMD indicates age-related macular degeneration.

Characteristics of participants and nonparticipants at 6-year follow-up are given in Table 1. Nonparticipants were divided into those who had died and those who lived but did not attend the examination clinic (eg, because of mass displacement in the period of postelection violence after the baseline study period) and/or those without a valid AMD assessment (eg, cataract obstructing a view of the retina). Compared with those followed up, participants who had died during follow-up were older and more likely to be female, have lower educational level, have higher systolic blood pressure, and have diabetes but lower body mass index. Compared with participants seen, those lost to follow-up were less likely to be Kikuyu or Kalenjin speakers, had lower levels of education, and were more likely to be from urban areas and from the highest or lowest socioeconomic quartile. Individuals seen at follow-up were less likely to have impaired vision at baseline (48 [3.7%]) compared with those who died before follow-up (30 [13.3%]) or those not seen at follow-up (97 [7.0%]).

**Table 1.** Baseline Characteristics of All 2900 Individuals With No AMD at Baseline According to Availability of AMD Status at 6-Year Follow-up^a^

Baseline Characteristic	Missing Values	Participants	Nonparticipants or Not Included in Analysis	
		Followed Up (n = 1282)	Not Followed Up^b^ (n = 1393)	Deceased (n = 225)
Age, mean (SD), y	0	60.7 (8.2)	61.8 (9.8)	68.5 (12.0)^c^
BP, mean (SD), mm Hg				
Systolic	26	138.4 (23.6)	139.3 (23.6)	143.3 (28.7)^c^
Diastolic	26	83.1 (13.0)	82.6 (13.1)	82.4 (16.0)
Random blood glucose level, mean (SD), mg/dL	79	5.2 (2.3)	5.2 (2.2)	5.6 (3.3)^c^
Sex				
Male	0	647 (50.5)	707 (50.8)	86 (38.2)
Female	0	635 (49.5)	686 (49.2)	139 (61.8)^c^
BMI^d^				
Underweight (<18.5)	29	139 (10.9)	188 (13.7)	52 (23.2)^c^
Normal (18.5-24.99)	0	648 (50.7)	674 (49.2)	113 (50.4)
Overweight (25-29.99)	0	307 (24.0)	321 (23.4)	40 (17.9)
Obese (≥30)	0	184 (14.4)	186 (13.6)	19 (8.5)
Vision status impaired, <6/12 better eye				
Normal	18	1233 (96.3)	1279 (93.0)^e^	195 (86.7)^c^
Impaired	0	48 (3.7)	97 (7.0)	30 (13.3)
Tribe				
Kikuyu	0	799 (62.3)	782 (56.1)^e^	152 (67.6)
Kalenjin	0	324 (25.3)	313 (22.5)	52 (23.1)
Other	0	159 (12.4)	298 (21.4)	21 (9.3)
Educational level				
None	21	119 (9.3)	163 (11.9)^e^	15 (6.7)^c^
Primary	0	345 (26.9)	432 (31.4)	91 (40.6)
Secondary	0	677 (52.8)	608 (44.3)	94 (42.0)
Higher	0	140 (10.9)	171 (12.4)	24 (10.7)
Residence				
Rural	0	937 (73.1)	763 (54.8)^e^	161 (71.6)
Urban	0	345 (26.9)	630 (45.2)	64 (28.4)
SES quartile				
Lower	34	266 (20.9)	324 (23.7)^e^	66 (29.5)
Middle lower	0	347 (27.2)	304 (22.2)	55 (24.6)
Middle upper	0	345 (27.1)	342 (25.0)	60 (26.8)
Upper	0	317 (24.9)	397 (29.0)	43 (19.2)
Smoker				
Never	15	861 (67.2)	980 (71.1)^e^	134 (59.6)
Former		104 (8.1)	124 (9.0)	22 (9.8)
Current		317 (24.7)	274 (19.9)	69 (30.7)
Alcohol use				
Never	23	524 (40.9)	512 (37.3)	61 (27.2)^c^
Former	0	549 (42.9)	595 (43.3)	116 (51.8)
Current	0	207 (16.2)	266 (19.4)	47 (21.0)

Abbreviations: AMD, age-related macular degeneration; BMI, body mass index; BP, blood pressure; SES, socioeconomic status.

^a^ Data are presented as number (percentage) of participants unless otherwise indicated.

^b^ Participants not followed up were alive, unknown, or had AMD status missing.

^c^ *P* < .05 for association between the baseline characteristic and the odds of dying during the follow-up period among all participants identified as non-AMD at baseline, excluding the group who were not followed up.

^d^ Calculated as weight in kilograms divided by height in meters squared.

^e^ *P* < .05 for association between the baseline characteristic and the odds of being followed up among all participants identified as non-AMD at baseline and not known to be deceased at follow-up.

In total, 1453 persons (50.1%) at risk for AMD were followed up after 6 years (Figure), and 1282 had data on AMD at follow-up. Of these, 202 developed early AMD, and no participants developed late AMD. The 6-year cumulative incidence of early AMD, after taking account of loss to follow-up by using IPW, was 164.2 per 1000 persons (95% CI, 136.7-195.9 per 1000 persons).

In addition, 366 participants with early AMD were at risk of progressing to late AMD at follow-up, of whom 173 were followed up and 142 had a valid AMD assessment (25 of 31 who did not have an AMD assessment had a lens opacity that obscured the retinal images). Two individuals with early AMD from the 142 at risk had developed late AMD at follow-up (Figure), giving a 6-year cumulative incidence of progression from early to late AMD of 24.5 per 1000 persons (95% CI, 5.0-111.7 per 1000 persons).

Of the 38 individuals with late AMD at baseline, 17 (44.7%) were followed up, 5 (13.2%) died, and 16 (42.1%) were not located for follow-up. Of the 17 individuals who were followed up, 4 (23.5%) did not have a valid AMD assessment (because of obstructing lens opacities), 11 (64.7%) remained classified as having late AMD, and 2 (11.8%) had a critical eye that was difficult to grade because of image quality but most likely had stable end-stage AMD. The visual status at baseline and follow-up is given in eTable 1 in the Supplement.

Cumulative incidence of AMD (≥80 vs 50-59 years of age: 243.8 per 1000 persons [95% CI, 115.8-442.4 per 1000 persons] vs 139.3 per 1000 persons [95% CI, 105.3-181.9 per 1000 persons]) strongly correlated with age (Table 2). The cumulative incidence of AMD was higher among women than men in each age group (197.0 per 1000 persons [95% CI, 156.7-244.7 per 1000 persons] vs 130.5 per 1000 persons [95% CI, 104.1-162.4 per 1000 persons]), with an overall 6-year cumulative incidence of 197 new cases per 1000 persons (95% CI, 157-245 per 1000 persons) among women compared with 131 new cases per 1000 persons (95% CI, 104-162 per 1000 persons) among men, giving an unadjusted risk ratio of 1.51 (95% CI, 1.14-2.00). For each increase in age category, the risk ratio was estimated to be 1.19 (95% CI, 1.00-1.42).

**Table 2.** Age- and Sex-Specific 6-Year Cumulative Incidence of Age-Related Macular Degeneration Among the Nakuru Eye Disease Cohort Study Participants

Age Group, y	Males		Females		Overall	
	No. of Cases/No. at Risk	Risk per 1000 at 6 Years (95% CI)	No. of Cases/No. at Risk	Risk per 1000 at 6 Years (95% CI)	No. of Cases/No. at Risk	Risk per 1000 at 6 Years (95% CI)
50-59	29/288	98.2 (64.8-146.1)	60/369	172.7 (129.4-226.6)	89/657	139.3 (105.3-181.9)
60-69	33/221	146.6 (103.6-203.4)	38/197	214.5 (152.0-293.7)	71/418	179.9 (142.8-224.3)
70-79	20/104	184.6 (121.3-270.9)	13/66	193.0 (109.3-318.0)	33/170	188.0 (138.2-250.6)
≥80	4/22	148.1 (51.0-360.0)	5/15	378.8 (132.6-708.7)	9/37	243.8 (115.8-442.4)
All ages	86/635	130.5 (104.1-162.4)	116/647	197.0 (156.7-244.7)	202/1282	164.2 (136.7-195.9)

On the basis of extrapolations of these results to census data and population estimates in 2015 (assuming incident cases annually is proportional to the cumulative incidence), we estimate that, in Kenya, 103 070 new cases of AMD (at any severity) develop every year in persons 50 years or older, of whom 65 720 (63.8%) are women (Table 3).

**Table 3.** Extrapolated Number of New Adults 50 Years and Older in Kenya Developing Age-Related Macular Degeneration per Year^a^

Age Group, y	Extrapolated No. (95% CI)		
	Males	Females	Overall
50-59	16 460 (10 860-24 500)	31 520 (23 630-41 360)	48 770 (36 890-63 700)
60-69	12 280 (8680-17 040)	21 800 (15 450-29 860)	33 450 (26 540-41 690)
70-79	6650 (4370-9750)	7720 (4370-12 710)	14 550 (10 700-19 400)
≥80	1520 (520-3710)	4550 (1590-8520)	5520 (2620-10 020)
All ages >50	38 280 (30 530-47 650)	65 720 (52 270-81 620)	103 070 (85 800-123 020)

^a^ On the basis of incidence data (adjusted for loss to follow-up) and estimates of the population in Kenya by age group in 2015.

Specific features of AMD that appear or regress during the study period were recorded (eTable 2 in the Supplement). Small drusen were noted to have a 6-year cumulative incidence of 59.1% (95% CI, 53.7%-64.3%) and a cumulative risk of 24.1% (95% CI, 20.6%-28.0%) for regression. Hyperpigmentation and hypopigmentation had a high cumulative risk of resolution during the 6-year follow-up period (hyperpigmentation: 77.0%; 95% CI, 59.5%-88.4%; hypopigmentation: 58.1%; 95% CI, 39.7%-74.4%) but a low incidence (hyperpigmentation: 3.5%; 95% CI, 2.5%-5.0%; hypopigmentation: 5.0%; 95% CI, 3.5%-7.1%).

Multivariable analysis of factors associated with incident AMD indicated an increasing incidence of AMD with older age (*P* for trend = .02), female sex (*P* = .001), and diabetes (*P* = .04) (Table 4).

**Table 4.** Age-Adjusted and Multivariable Analysis of Baseline Covariables and Incident AMD in the Nakuru Eye Disease Cohort Study Sample of 1282 Individuals

Covariable	No. at Risk of AMD^a^	No. With Incident AMD^b^	Risk per 1000 at 6 Years (95% CI)	Age-Adjusted Risk Ratio (95% CI)	Baseline *P* Value	Multivariable-Adjusted Risk Ratio (95% CI)^c^	Baseline *P* Value
Age group, y							
50-59	657	89	139.3 (105.3-181.9)	1 [Reference]	.16	1 [Reference]	.07
60-69	418	71	179.9 (142.8-224.3)	1.3 (1.0-1.7)		1.3 (1.0-1.7)	
70-79	170	33	188.0 (138.2-250.6)	1.4 (0.9-1.9)		1.5 (1.0-2.1)	
≥80	37	9	243.8 (115.8-442.4)	1.8 (0.8-3.7)		1.8 (0.9-3.5)	
Sex							
Male	635	86	130.5 (104.1-162.4)	1 [Reference]	.002	1 [Reference]	.001
Female	647	116	197.0 (156.7-244.7)	1.6 (1.2-2.1)		1.6 (1.2-2.1)	
BMI^d^ (4 missing values)							
Underweight (<18.5)	139	25	191.3 (127.2-277.4)	1 [Reference]	.84	NA	NA
Normal (18.5-24.99)	648	98	160.1 (129.9-195.7)	0.8 (0.5-1.3)		NA	
Overweight (25-29.99)	307	49	159.4 (113.0-220.1)	0.9 (0.6-1.4)		NA	
Obese (≥30)	184	30	169.1 (119.0-234.6)	0.9 (0.6-1.6)		NA	
Location							
Rural	937	146	160.0 (131.9-192.8)	1 [Reference]	.55	NA	NA
Urban	345	56	171.2 (116.8-244.0)	1.1 (0.8-1.7)		NA	
SES quartile (7 missing values)							
Lower	266	50	216.0 (154.0-294.2)	1 [Reference]	.07	NA	NA
Lower middle	347	43	128.5 (94.4-172.4)	0.6 (0.4-0.9)		NA	
Upper middle	345	59	169.1 (128.1-219.8)	0.8 (0.5-1.3)		NA	
Upper	317	48	148.5 (105.9-204.4)	0.7 (0.5-1.1)		NA	
Smoker							
Never	861	142	173.6 (141.6-211.1)	1 [Reference]	.12	NA	NA
Former	104	9	83.5 (39.5-167.9)	0.5 (0.2-1.0)		NA	
Current	317	51	165.7 (125.5-215.7)	0.9 (0.7-1.2)		NA	
Hypertension (2 missing values)							
No	664	95	149.1 (113.3-193.6)	1 [Reference]	.37	NA	NA
Yes	616	106	178.0 (143.1-219.2)	1.2 (0.8-1.6)		NA	
Diabetes (1 missing value)							
No	1223	188	157.8 (131.7-187.9)	1 [Reference]	.07	1 [Reference]	.04
Yes	58	14	263.9 (136.3-448.9)	1.7 (1.0-2.8)		1.7 (1.0-2.8)	
Alcohol use (2 missing values)							
Never	524	85	171.6 (133.7-217.5)	1 [Reference]	.59	NA	NA
Former	549	81	155.2 (117.5-202.2)	0.8 (0.6-1.2)		NA	
Current	207	36	169.6 (116.3-240.6)	1.0 (0.6-1.4)		NA	
Ethnic group							
Kikuyu	799	126	164.7 (130.5-205.6)	1 [Reference]	.51	NA	NA
Kalenjin	324	46	148.5 (107.4-201.7)	0.9 (0.6-1.3)		NA	
Other	159	30	184.4 (119.2-274.3)	1.2 (0.8-2.0)		NA	
Educational level (1 missing value)							
No education	119	12	111.3 (58.2-202.5)	1 [Reference]	.16	NA	NA
Primary	345	66	215.8 (161.8-281.7)	1.8 (0.9-3.4)		NA	
Secondary	677	104	152.8 (123.7-187.4)	1.3 (0.7-2.5)		NA	
College or university	140	20	128.5 (80.7-198.4)	1.2 (0.5-2.6)		NA	

Abbreviations: AMD, age-related macular degeneration; BMI, body mass index; NA, not applicable; SES, socioeconomic status.

^a^ At risk indicates no AMD at baseline.

^b^ Incident AMD indicates early or late AMD at follow-up.

^c^ For multivariable analysis, an initial model was fitted that included those variables associated with outcome in age-adjusted analysis (using a Wald test threshold *P* < .05 to indicate association). A backward stepwise approach was then applied to obtain a final multivariable model, removing variables with *P* > .05 one by one.

^d^ Calculated as weight in kilograms divided by height in meters squared.

Of the 234 individuals in the cohort who developed incident vision impairment, 162 (69.2%) had an available AMD assessment at follow-up. Of the 162 individuals, 52 (32.1%) had AMD, and 3 of these patients were classified as blind. It was not possible to infer whether vision loss was attributable solely to AMD or a combination of other ocular comorbidities. Change in vision category from baseline to follow-up in all those with a valid AMD status at baseline and follow-up is given in eTable 3 in the Supplement.

A total of 202 participants in the cohort developed incident AMD, of whom 192 (95.0%) had normal vision at baseline. A total of 27 (14.1%) of the 192 developed visual impairment by follow-up. A total of 1080 participants did not develop AMD, with 1040 (96.3%) in this group having normal vision at baseline. Of these 1040 individuals, 83 (8.0%) developed vision impairment.

## Discussion

The Nakuru Eye Disease Cohort Study provides longitudinal data on AMD in sub-Saharan Africa from a population-based cohort. Although there is 50% loss to follow-up and few cases of late AMD (resulting in wide CIs for that outcome), there are limited data on these outcomes from this region. With those caveats in mind, during 6 years, 1 in 6 adults 50 years or older developed early manifestations of AMD, with women having a higher incidence than men. Increasing age was strongly related to the prevalence and incidence of AMD. Most incident cases of AMD were defined on the basis of the development of large drusen (>64 μm). Late AMD was infrequent at baseline, and consistent with this pattern, only 2 cases of incident late AMD were found at follow-up. Both incident cases developed in individuals with early AMD at baseline, and no case was identified that progressed from no AMD to late AMD.

Our data estimate a higher incidence of AMD than other (non-African) cohort studies of eye disease (eTable 4 in the Supplement). A likely explanation is that the Nakuru cohort includes only persons 50 years or older, similar to the next 2 highest cumulative annual incidence estimates, which were also from samples of older individuals in Copenhagen^6^ and Reykjavik.^11^ Furthermore, in the Nakuru study, there was a higher incidence of early AMD and a lower incidence of late AMD compared with other populations. This observation is consistent with the baseline finding, which indicated a comparatively high prevalence of early AMD but low prevalence of late AMD.^5^ The 2 participants who progressed from early to late AMD were older than 80 years; the reduced number of individuals in this age group in the Nakuru cohort may explain the low incidence of late AMD because age is the leading risk factor for incident AMD.

A high prevalence of early AMD at baseline and a high incidence of early AMD at follow-up may suggest that the population under investigation has a higher risk of developing AMD. The relatively high prevalence and incidence may possibly be attributed to greater UV light exposure, earlier biological aging, greater genetic predisposition, or greater susceptibility to inflammatory processes, which have been attributed to AMD.^19^ The proportion of persons with vision loss attributable to AMD is relatively low because overall vision loss primarily attributable to conditions such as cataract is largely under control in more developed health care systems. Of those with AMD, 14% developed vision impairment during the 6-year study period compared with 9% during 14 years in a well-developed health care system in Copenhagen. However, vision impairment cannot be attributed to AMD alone.

### Strengths and Limitations

This study was conducted under challenging circumstances with limited infrastructure. It provides independently graded, digital image–based analysis of AMD in an East African cohort that is diverse and largely representative of the population from which it was sampled. Detailed ophthalmic, demographic, and anthropometric assessment of each participant has enabled valuable analyses of associations and risk factors.

Despite these strengths, limitations of the current study may have contributed to an underestimation of the true incidence of AMD and AMD lesions. First, the main limitation of our study is the large loss of participants at follow-up. Of the 4414 persons who participated in the baseline study, only 2171 persons participated in the follow-up examination, of whom 1424 had gradable images at baseline and follow-up, with most being excluded from analysis as a result of camera failure at baseline, lack of electricity access, and/or ungradable images attributable to media opacities. Only those who had gradable images at both time points were included in the analysis. A complete case record analysis was conducted without weighting for loss to follow-up (eTable 5 in the Supplement), with results similar to those using the IPW modeling (Table 2), with a possible underestimation of the incidence in women when loss to follow-up is not taken into account.

Second, changes in procedures between the baseline and follow-up examination (different retinal camera) may have introduced bias. Change in cameras may have caused images with different color profiles and saturation levels, resulting in different abilities to detect AMD features (eg, drusen and pigment). Furthermore, the lack of stereophotographs meant cases of retinal elevation may have been overlooked. These factors may have resulted in bias toward an underestimation of the incidence of subtle early AMD lesions, such as small drusen, or an overestimation of pigmentation attributed to AMD. Comparison images between cameras of the same study participants at baseline and follow-up are given in eTable 6 in the Supplement. Overall image quality was considered to be equivalent at the 2 time points in those with clear media (ie, no lens or corneal opacity).

Third, survival bias may have caused an underestimation of the true incidence of late AMD if those who died before the follow-up had experienced advanced AMD lesions after the first examination, and it is possible that those with worsening disease were more likely to attend follow-up visits. The low prevalence of late AMD at baseline and low incidence can, in part, be attributed to a lack of older individuals in this study population, with an expected shorter life span than other populations with data on AMD incidence. Classification bias may also have contributed to the estimates, and histologic studies would be required to confirm whether the manifestations being attributed to AMD are consistent with those in other populations.

## Conclusions

We estimate that, in Kenya, more than 100 000 new cases of AMD, mainly early AMD, will develop every year in persons 50 years and older, although a 50% loss to follow-up and wide CIs for progression to late AMD limit definitive conclusions from these findings. The AMD in this population was found to be phenotypically different from that in a prior study.^5^ However, because the relatively high incidence was restricted to occurrence of early AMD, the high incidence of early AMD may not have major implications for clinical practice given the low number of individuals with associated visual loss.

## Notes

Supplement. **eMethods.** Ethics Statement, Sampling Strategy, and Inverse Probability Weighting **eTable 1**. Change in Presenting Visual Acuity Category in Those With Late AMD at Baseline in Those With an AMD Status Available at Both Time Points **eTable 2.** Incidence of Appearance and Regression of Individual Features of AMD Between Baseline and Follow-up **eTable 3.** Change in Presenting Visual Acuity Category From Baseline to Follow-up in Cohort With Visual Acuity Data and AMD Status Available at Both Time Points **eTable 4.** Population-Based Cohort Studies of AMD **eTable 5.** Unweighted for Missing Data (Complete Case Records Only) Age-and Sex-Specific 6-Year Cumulative Incidence of AMD Among the Nakuru Eye Disease Cohort Study Participants **eTable 6.** Side by Side Image Comparison Between Baseline and Follow-up
