# Supplementary material for: Six-Year Incidence and Progression of Age-Related Macular Degeneration in Kenya: Nakuru Eye Disease Cohort Study
Source: JAMA Ophthalmol. 2017 Jun 8;135(6):631–8. doi: 10.1001/jamaophthalmol.2017.1109 (PMC5710264; doi:10.1001/jamaophthalmol.2017.1109)
Supplement: Supplement. — eMethods. Ethics Statement, Sampling Strategy, and Inverse Probability Weighting eTable 1. Change in Presenting Visual Acuity Category in Those With Late AMD at Baseline in Those With an AMD Status Available at Both Time Points eTable 2. Incidence of Appearance and Regression of Individual Features of AMD Between Baseline and Follow-up eTable 3. Change in Presenting Visual Acuity Category From Baseline to Follow-up in Cohort With Visual Acuity Data and AMD Status Available at Both Time Points eTable 4. Population-Based Cohort Studies of AMD eTable 5. Unweighted for Missing Data (Complete Case Records Only) Age-and Sex-Specific 6-Year Cumulative Incidence of AMD Among the Nakuru Eye Disease Cohort Study Participants eTable 6. Side by Side Image Comparison Between Baseline and Follow-up [file jamaophthalmol-135-631-s001.pdf]

Supplementary Online Content

Bastawrous A, Mathenge W, Peto T, et al. Six-year incidence and progression of age-related macular degeneration in Kenya: Nakuru Eye Disease Cohort Study. *JAMA Ophthalmol.* Published online May 11, 2017. doi:10.1001/jamaophthalmol.2017.1109

eMethods. Ethics Statement, Sampling Strategy, and Inverse Probability Weighting

eTable 1. Change in Presenting Visual Acuity Category in Those With Late AMD at Baseline in Those With an AMD Status Available at Both Time Points

eTable 2. Incidence of Appearance and Regression of Individual Features of AMD Between Baseline and Follow-up

eTable 3. Change in Presenting Visual Acuity Category From Baseline to Follow-up in Cohort With Visual Acuity Data and AMD Status Available at Both Time Points

eTable 4. Population-Based Cohort Studies of AMD

eTable 5. Unweighted for Missing Data (Complete Case Records Only) Age-and Sex-Specific 6-Year Cumulative Incidence of AMD Among the Nakuru Eye Disease Cohort Study Participants

eTable 6. Side by Side Image Comparison Between Baseline and Follow-up

eReferences

This supplementary material has been provided by the authors to give readers additional information about their work.

© 2017 Bastawrous A et al. *JAMA Ophthalmol.*

eMethods. Ethics Statement, Sampling Strategy, and Inverse Probability Weighting

Ethics Statement

The London School of Hygiene & Tropical Medicine (LSHTM) Ethics committee and the African Medical Research Foundation (AMREF) granted ethical approval for the study and by the Provincial Medical Officer for Nakuru County. Written approval was sought from the administrative heads in each cluster, usually the village chief. All participants gave written or thumbprint consent to participate. People requiring medical treatments were referred to the appropriate centre.

Sampling Strategy and Recruitment

The study baseline fieldwork was carried out at baseline between January 2007 and November 2008. The follow-up study took place between October 2012 and March 2014.

At baseline, 100 clusters were selected across Nakuru County with a probability proportional to the size of the population using the electoral roll as the sampling frame. A cluster was defined as the area served by a polling station. Households were selected within clusters using a modified compact segment sampling method.[1] Each cluster was divided into segments so that each segment included approximately 50 people aged ≥50 years. One segment was selected at random, and all eligible people were included sequentially until 50 had been examined.

The sample size of 5000 people at baseline (2007-2008) was sufficient to estimate a prevalence of AMD of 3.0% among those aged ≥50 years, with a required precision of 0.5%, 95% confidence, a design effect to account for clustering of 1.5, and a response rate of 90%. (Epi Info 6.04, Centers for Disease Control and Prevention, Atlanta, GA). In total, 4,381 participants were recruited at baseline (response rate 81%).

© 2017 Bastawrous A et al. *JAMA Ophthalmol.*

All participants were invited to attend an examination clinic at a central location within the cluster (see below).

Follow-up

One week before the follow-up examination clinic was carried out a field officer studied the maps of the village including GPS coordinates recorded at baseline and made phone contact with the village chief or guide to arrange a planning visit. A list of study participants were given to the chief and a local village guide was recruited to assist locating the study participants. On the day prior to the examination clinic, a study team visited homes of baseline participants and confirmed their identity using National Identity cards and invited them to attend the examination clinic the following day.

On the examination day, the advance team confirmed the identity of participants against data from baseline (age, date of birth, name, and identity cards). In cases of uncertain identity, confirmation was made based on retinal examination verified by comparison of retinal photos with baseline photo (n=12).

Visual Acuity

All participants underwent visual acuity (VA) testing on each eye separately at four meters using a reduced LogMAR tumbling 'E' chart[2] in a well illuminated area as described elsewhere.[3,4] Presenting VA was defined as the number of letters read correctly without glasses if the participant did not have glasses or with glasses if they had them.

All participants underwent Autorefraction and those with a presenting acuity of <24 LogMAR letters (<20/40 Snellen Equivalent) had a corrected VA assessed in addition to presenting (uncorrected, under corrected or corrected). More detailed methodology is available elsewhere.[5]

© 2017 Bastawrous A et al. *JAMA Ophthalmol.*

Fundus photography

The participants had two non-stereoscopic digital 45^0 fundus photographs taken per eye by an ophthalmic clinical officer using a TRC-NW6S Non-Mydriatic Retinal Camera with 10 megapixel Nikon D80 (Top Con®) at baseline and a DRS CentreVue+ (Haag-Streit) Retinal Camera at follow-up. One image was centred on the optic disc while the other was centred on the macula. The digital images were forwarded to the Retinal Grading Centre at Moorfields Eye Hospital Reading Centre (MEHRC) London for grading and confirming the clinical diagnosis of posterior segment disease.

Questionnaire and anthropometry

Detailed interviews were undertaken in the local language covering demographic details, information on risk factors, socio-economic status (SES) and full past medical history. SES was evaluated using a continuous asset score, which was produced for each participant, using a scoring system derived through principal component analysis in an earlier study in this setting.[6,7] The scale included assessment of 17 asset items and five measures of household characteristics.

A nurse recorded the blood pressure of participants three times on the right arm of the participant, at least five minutes apart after an initial period of five minutes of rest using the Omron digital automatic monitor (model HEM907). Weight was measured to the nearest kilogram using standard scales (Seca 761 scales) after the participant had removed all heavy clothing and shoes. Height was measured to the nearest centimetre while the participant stood without shoes using a standardized stadiometer (Leicester Height Measure). For weight and height the average of two readings was recorded. Waist and hip circumferences were measured with a tape to the nearest centimetre.

© 2017 Bastawrous A et al. *JAMA Ophthalmol.*

Image Grading

The senior grader (NS) graded all images for the presence of AMD. All images were first categorized for quality as excellent, good, fair, borderline and ungradeable. All questionable lesions and all eyes classified as having late-stage AMD were adjudicated by the MEHRC clinician (TP). Any lesions considered to be due to other causes such as myopia and inflammatory disease were not graded for AMD, and these were also verified by TP. The adjudicator also graded 5% of randomly selected images to ensure quality control. Data were single entered onto Excel and checked for consistency by an independent data monitor from MEHRC who was not involved in the study.

Data Handling & Statistical Analyses Methods

Data entry

Image data were double entered into a specially developed dataset (EpiData Entry v2.1). Consistency checks were performed each evening and inconsistencies corrected the same day.

Data analysis

Individuals in the study who were classified as AMD free at baseline were defined as being at risk of developing AMD during the follow-up period of the study.

Inverse Probability Weighting

Of the 2900 individuals at risk of AMD at baseline, 225 were confirmed as deceased during the follow up period. This left 2675 individuals eligible for follow up. Of these 1393 (52%) did not have a valid AMD status at follow up, leaving 1282 individuals eligible for inclusion in the incidence study. To take account for any bias due to this loss to follow up, inverse probability weights were estimated for individuals who

© 2017 Bastawrous A et al. *JAMA Ophthalmol.*

were not confirmed as deceased, then this weighting was applied to the estimates of incidence.

Variables found to be associated with loss to follow up were: age group, residence, socio-economic status, smoking status, alcohol status, tribe, education level and baseline diabetes status. Of those that were followed up, socio-economic status was missing for 7 individuals. So these individuals were excluded from the weighted estimates, as the number missing was small and socio-economic status was a strong predictor of missingness.

© 2017 Bastawrous A et al. *JAMA Ophthalmol.*

eTable 1. Change in Presenting Visual Acuity Category in Those With Late AMD at Baseline in Those With an AMD Status Available at Both Time Points

Follow-up							
Baseline		**Normal**	**Mild VI**	**Mod VI**	**Severe VI**	**Blind**	**Total**
	**Normal**	2	4	1	0	0	7
	**Mild VI**	0	0	0	0	0	0
	**Mod VI**	0	0	4	2	3	9
	**Severe VI**	0	0	0	0	0	0
	**Blind**	0	0	0	0	1	1
	**Total**	2	4	5	2	4	17

The proportion in brackets after each number is the proportion that report either baseline or incident AMD (total N=17).

© 2017 Bastawrous A et al. *JAMA Ophthalmol.*

eTable 2. Incidence of Appearance and Regression of Individual Features of AMD Between Baseline and Follow-up

	Feature measured at baseline and follow up (n)	Feature absent at baseline (n)	Feature present at follow-up (n)	6 year cumulative incidence of feature appearance (Adjusted for LTFU using IPW)	Feature present at baseline (n)	Feature absent at follow-up (n)	6 year cumulative incidence of feature regression (Adjusted for LTFU using IPW)
Small drusen	1220	446	261	59.1% (53.7%,64.3%)	774	188	24.1% (20.6%,28.0%)
Large drusen	1134	1039	196	19.6% (16.3%,23.5%)	95	8	6.8% (3.3%,13.5%)
GA	1083	1077	1	0.3% (0.0%,2.0%)	6	1	19.2% (0.7%,89.2%)
CNV	1083	1075	2	0.2% (0.0%,0.7%)	8	2	24.6% (3.4%,75.4%)
Hyperpigmentation	1090	1050	36	3.5% (2.5%,5.0%)	40	30	77.0% (59.5%,88.4%)
Hypopigmentation	1088	1053	48	5.0% (3.5%,7.1%)	35	21	58.1% (39.7%,74.4%)
RPE	1081	1080	0	-	1	1	100.0%

© 2017 Bastawrous A et al. *JAMA Ophthalmol.*

detachment							

LTFU: Loss to follow-up, IPW: Inverse Probability Weighting

© 2017 Bastawrous A et al. *JAMA Ophthalmol.*

eTable 3. Change in Presenting Visual Acuity Category From Baseline to Follow-up in Cohort With Visual Acuity Data and AMD Status Available at Both Time Points

Follow-up							
		Normal	Mild VI	Mod VI	Severe VI	Blind	Total
	**Normal**	1,058 (21.6%)	153 (30.7%)	103 (35.0%)	0 (N/A)	3 (66.7%)	1,317 (23.8%)
	**Mild VI**	13 (23.1%)	16 (31.3%)	22 (31.8%)	0 (N/A)	0 (N/A)	51 (29.4%)
Baseline	**Mod VI**	9 (33.3%)	9 (55.6%)	34 (41.2%)	7 (42.9%)	1 (0.0%)	60 (41.7%)
	**Severe VI**	0 (N/A)	0 (N/A)	2 (50.0%)	0 (N/A)	0 (N/A)	2 (50.0%)
	**Blind**	0 (N/A)	0 (N/A)	1 (100.0%)	0 (N/A)	4 (50.0%)	5 (60.0%)
	**Total**	1,080 (21.7%)	178 (32.0%)	162 (36.4%)	7 (42.9%)	8 (50.0%)	1,435 (24.9%)

The proportion in brackets after each number is the proportion that report either baseline or incident AMD (total N=1,435)

© 2017 Bastawrous A et al. *JAMA Ophthalmol.*

eTable 4. Population-Based Cohort Studies of AMD

Study	Location	Year commenced	Years of Follow up	No of participants	Age at Baseline	Cumulative incidence of Early AMD (%)	Cumulative annual incidence of Early AMD (%)*	Cumulative (study period) Incidence of Late AMD (%)**	Reference
Nakuru	Kenya	2007	Baseline<br>6	4414<br>2171	50+	16.4	2.9	0.2	This paper
Studies of equivalent age groups									
Blue Mountain Eye Study	Australia	1992	Baseline<br>5<br>10	3654<br>2335<br>1952	49+	14.1	1.4	3.7	[8]
Reykjavik Eye Study	Iceland	1996	Baseline<br>5	1045<br>846	50+	10.7	2.1		[9]
Studies of different age groups									
Beaver Dam Eye Study	USA	1988	Baseline<br>5<br>10<br>15	4926<br>3684<br>2764<br>2119	43-86	12.1<br>14.3	1.0	2.1<br>3.1	[10-12]
Copen	Den	1986	Base	946	60-				[13]

© 2017 Bastawrous A et al. *JAMA Ophthalmol.*

hagen City Eye Study	mark		line 14	359	80	31.5	2.3	14.8	
Barbados Eye Study	Barbados	1987	Baseline<br>4<br>9	4631<br>3427<br>2793	40+	 5.2<br>12.6	 <br>1.4	 Negligible<br>0.7	14,15
Hisayama Study	Japan	1998	Baseline<br>5<br>9	1482<br>961<br>1401(>40yrs)	40+	 8.5<br>10.0	 <br>1.1	 0.8<br>1.4	16,17
Los Angeles Latino Eye Study	USA	2000	Baseline<br>4	6357<br>4658	40+	 7.5	 1.9	 0.2	18

*Annual cumulative incidence is calculated as the overall cumulative incidence divided by the number of years of follow up, where more than one follow-up visit was conducted, the longest one is used.

**Incident Late AMD considered as those without Late AMD (no AMD or Early AMD at baseline).

© 2017 Bastawrous A et al. *JAMA Ophthalmol.*

eTable 5. Unweighted for Missing Data (Complete Case Records Only) Age-and Sex-Specific 6-Year Cumulative Incidence of AMD Among the Nakuru Eye Disease Cohort Study Participants

	Male		Female		Overall	
Age Group (years)	N (Cases / at risk)	Risk per 1,000/6yrs (95%CI)*	N	Risk per 1,000/6yrs (95%CI)*	N	Risk per 1,000/6yrs (95%CI)*
50-59	29 / 288	100.7(66.0,150.7)	60 / 369	162.6(123.5,211.1)	89 / 657	135.5(103.5,175.3)
60-69	33 / 221	149.3(107.2,204.2)	38 / 197	192.9(141.9,256.7)	71 / 418	169.9(137.6,207.9)
70-79	20 / 104	192.3(128.5,277.7)	13 / 66	197.0(113.2,320.4)	33 / 170	194.1(144.8,255.2)
80+	4 / 22	181.8(70.6,394.0)	5 / 15	333.3(137.5,610.6)	9 / 37	243.2(131.3,406.0)
All ages	86 / 635	135.4(108.0,168.5)	116 / 647	179.3(145.3,219.2)	202 / 1282	157.6(132.3,186.6)

© 2017 Bastawrous A et al. *JAMA Ophthalmol.*

eTable 6. Side by Side Image Comparison Between Baseline and Follow-up

Baseline – TopCon NRW6	Followup – Haag Streit DRS CentreVue
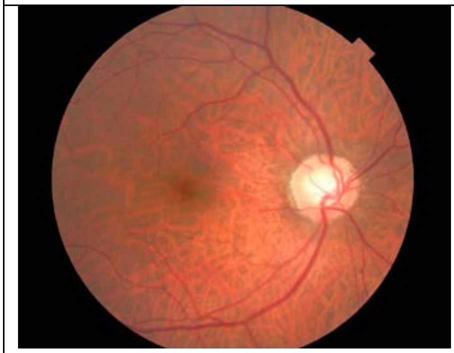	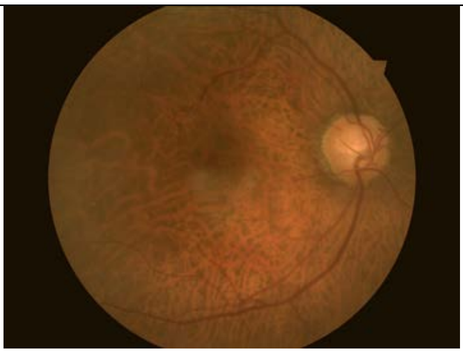
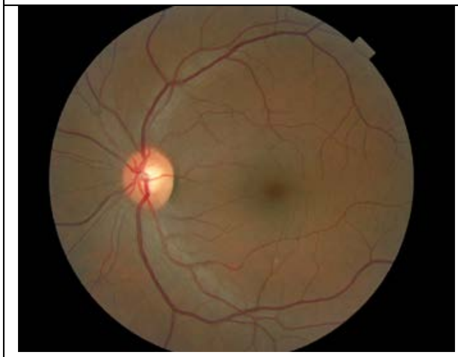	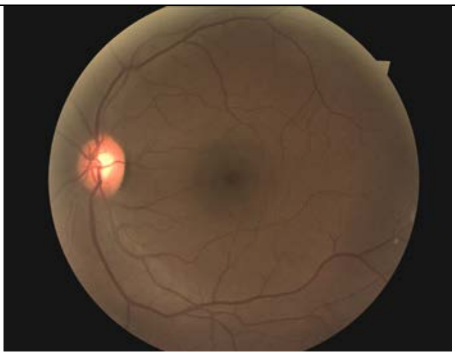

© 2017 Bastawrous A et al. *JAMA Ophthalmol.*

eReferences

1. Turner AG, Magnani RJ, Shuaib M. A not quite as quick but much cleaner alternative to the Expanded Programme on Immunization (EPI) Cluster Survey design. *Int J Epidemiol*. 1996;25(1):198-203.
2. Rosser DA, Laidlaw DA, Murdoch IE. The development of a “reduced logMAR” visual acuity chart for use in routine clinical practice. *Br J Ophthalmol.* 2001;85(4):432-436.
3. Dineen B, Gilbert CE, Rabiu M, et al. The Nigerian national blindness and visual impairment survey: rationale, objectives and detailed methodology. *BMC Ophthalmol.* 2008;8:17.
4. Bastawrous A, Rono HK, Livingstone IA, et al. Development and validation of a smartphone-based visual acuity test (peek acuity) for clinical practice and community-based fieldwork. *JAMA Ophthalmol.* 2015;133(8):930-937.
5. Bastawrous A, Mathenge W, Peto T, et al. The Nakuru eye disease cohort study: methodology & rationale. *BMC Ophthalmol.* 2014;14:60.
6. Kuper H, Polack S, Eusebio C, Mathenge W, Wadud Z, Foster A. A case-control study to assess the relationship between poverty and visual impairment from cataract in Kenya, the Philippines, and Bangladesh. *PLoS Med.* 2008;5(12):e244.
7. Polack S, Kuper H, Mathenge W, Fletcher A, Foster A. Cataract visual impairment and quality of life in a Kenyan population. *Br J Ophthalmol.* 2007;91(7):927-932.
8. Tan JS, Mitchell P, Kifley A, Flood V, Smith W, Wang JJ. Smoking and the long-term incidence of age-related macular degeneration: the Blue Mountains Eye Study. *Arch Ophthalmol.* 2007;125(8):1089-1095.
9. Jonasson F, Arnarsson A, Peto T, Sasaki H, Sasaki K, Bird AC. 5-Year incidence of age-related maculopathy in the Reykjavik Eye Study. *Ophthalmology.* 2005;112(1):132-138.
10. Klein R, Klein BE, Jensen SC, Meuer SM. The five-year incidence and progression of age-related maculopathy: the Beaver Dam Eye Study. *Ophthalmology*. 1997;104(1):7-21.
11. Klein R, Klein BE, Knudtson MD, Meuer SM, Swift M, Gangnon RE. Fifteen-year cumulative incidence of age-related macular degeneration: the Beaver Dam Eye Study. *Ophthalmology*. 2007;114(2):253-262.
12. Klein R, Klein BE, Tomany SC, Meuer SM, Huang GH. Ten-year incidence and progression of age-related maculopathy: the Beaver Dam Eye Study. *Ophthalmology*. 2002;109(10):1767-1779.
13. Buch H, Nielsen NV, Vinding T, Jensen GB, Prause JU, la Cour M. 14-Year incidence, progression, and visual morbidity of age-related maculopathy: the Copenhagen City Eye Study. *Ophthalmology*. 2005;112(5):787-798.
14. Leske MC, Wu SY, Hyman L, Hennis A, Nemesure B, Schachat AP; Barbados Eye Studies Group. Four-year incidence of macular changes in the Barbados Eye Studies. *Ophthalmology*. 2004;111(4):706-711.
15. Leske MC, Wu SY, Hennis A, et al; Barbados Eye Studies Group. Nine-year incidence of age-related macular degeneration in the Barbados Eye Studies. *Ophthalmology*. 2006;113(1):29-35.

© 2017 Bastawrous A et al. *JAMA Ophthalmol.*

16. Yasuda M, Kiyohara Y, Hata Y, et al. Nine-year incidence and risk factors for age-related macular degeneration in a defined Japanese population: the Hisayama study. *Ophthalmology*. 2009;116(11):2135-2140.
17. Miyazaki M, Kiyohara Y, Yoshida A, Iida M, Nose Y, Ishibashi T. The 5-year incidence and risk factors for age-related maculopathy in a general Japanese population: the Hisayama study. *Invest Ophthalmol Vis Sci.* 2005;46(6):1907-1910.
18. Varma R, Foong AW, Lai MY, Choudhury F, Klein R, Azen SP; Los Angeles Latino Eye Study Group. Four-year incidence and progression of age-related macular degeneration: the Los Angeles Latino Eye Study. *Am J Ophthalmol.* 2010;149(5):741-751.

© 2017 Bastawrous A et al. *JAMA Ophthalmol.*
